# Video-based interviewing in medicine: protocol for a scoping review

**DOI:** 10.1186/s13643-020-01484-6

**Published:** 2020-09-25

**Authors:** Rajajee Selvam, Richard Hu, Reilly Musselman, Isabelle Raiche, Husein Moloo

**Affiliations:** 1 Department of Surgery, The Ottawa Hospital - Civic Campus, CPC Building - Room 300, 1053 Carling Avenue, Ottawa, Ontario K1Y 1J8 Canada; 2 The Ottawa Hospital Research Institute, Ottawa, Ontario Canada

**Keywords:** Video-based interviews, Videoconferencing, Personnel selection, Climate change

## Abstract

**Background:**

Careers in healthcare involve an extensive interview process as transitions are made from one level of training to the next. For physicians, interviews mark the gateway from entrance into medical school, acceptance into residency, fellowships, and subsequent job opportunities. Previous literature outlining the costs associated with face-to-face interviews and concerns regarding the climate crisis has triggered an interest in video-based interviews. Barriers to transitioning away from in-person interviews include concerns regarding lack of rapport between applicants and interviewers, and applicants being less able to represent themselves. In a new era ushered in by COVID where many of us have utilized virtual meetings more than any prior time both personally and for work, we wanted to consolidate the current literature on the use of video-based interviews in healthcare and summarize the findings.

**Methods:**

A scoping review will be conducted to explore the benefits and limitations of video-based interviews for both applicants and interviewers within healthcare fields, as well as the perceived barriers associated with transitioning away from face-to-face interviews. The scoping review methodology outlined by Arksey and O’Malley will be implemented. The search strategy developed by the authors in collaboration with an academic health sciences librarian will be conducted across four electronic databases (Embase, MEDLINE, Cochrane Central, and PsycInfo) and supplemented by a review of the grey literature and reference lists of included studies. The study selection process will be documented using the PRISMA flow diagram, and reasons for exclusion following full-text review will be recorded. The extracted data will be analyzed using quantitative and qualitative analysis.

**Discussion:**

Despite previous literature on the costs associated with face-to-face interviews, there has been hesitancy with transitioning to video-based interviews due to concerns of lack of rapport between applicants and interviewers, and applicants being less able to represent themselves. While these limitations have been explored in previous studies, a succinct review of the current literature to guide the effective restructuring of the interview process is lacking. With our scoping review, we hope to fill this gap in the literature to better understand barriers to transitioning from face-to-face interviews and directions for future research.

## Background

Whether applying to medical school, residency programs, fellowships, or eventually for the role of a staff physician, interviews play a pivotal role for both applicants and programs in guiding their decision-making.

The financial costs associated with the interview process have been previously explored. Survey studies have reported that medical students applying to residency often needed to borrow money to cover the costs associated with interviews, with some medical students declining interview offers on the basis of financial costs [1, 2]. These costs include those associated with application fees, travel, and accommodation for interviews, as well as costs associated with completing elective rotations at other programs [1–3]. In addition to these financial costs, face-to-face interviews are accompanied by the opportunity costs of time taken away from clinical and academic activities. Studies have reported that general surgery residents applying for fellowship positions missed about 7 days of training days, which have been perceived by applicants and program directors to disrupt their program, decrease the time available for patient care, and increase workload for other residents [4]. A similar opportunity cost applies to interviewers who need to take time away from their clinical practice to participate in the interview process [3].

A less well-studied impact of face-to-face interviews is the environmental impact of long-haul flights, trains, and other forms of transportation. While these factors have not been directly evaluated as they apply to interview processes in medicine, there has been a recent emphasis on the role that healthcare practitioners play on climate change [5, 6]. Solomon and LaRocque highlight the significant contribution to greenhouse gasses made by the healthcare sector and have posited that healthcare professionals have an ethical obligation to address climate change [5]. Harmer and colleagues suggest that the World Health Organization (WHO) declares climate change as a public health emergency. Similar to infectious disease outbreaks, climate change has both direct and indirect effects on mortality, impacts health systems on a global scale, and requires urgent response to mitigate its ongoing detrimental effects [6]. As physicians with an obligation to do no harm, it is prudent to identify and implement measures to minimize our carbon footprint.

In light of the aforementioned environmental, financial, and opportunity costs associated with face-to-face interviews, some programs have attempted to transition towards video-based interviewing with differing results [7–11]. In a study of applicants given the option between video-based or face-to-face interviews, video-based interviews were primarily chosen when there were financial limitations, travel concerns, or interview conflicts [11]. While video-based interviews were found to be a viable alternative to face-to-face interviews in a pilot study of medical students applying to the anesthesiology residency program, other studies view video-based interviews as an effective adjunct rather than a replacement to the traditional face-to-face interview [9, 11]. Barriers to transitioning to video-based interviews have mainly been due to concerns of less rapport and being less effective in allowing applicants to represent themselves [9].

Given this recent trend towards cost-minimization within the interview process and the need for active measures to combat climate change, an opportunity was identified to consolidate the current literature on video-based interviews. To our knowledge, there has not been a formal review of the literature on this topic. The objectives of this review will be to evaluate the extent of previous research in this field and summarize research findings to help guide future research. Specifically, our review will focus on the benefits and limitations of video-based interviews for both applicants and programs in healthcare fields, as well as the perceived barriers associated with transitioning from face-to-face interviews.

## Methods/design

### Design

A scoping review will be undertaken to summarize the benefits and limitations of video-based interviews, as well as the barriers to transitioning from face-to-face interviews. A scoping review, rather than a systematic review, has been chosen as we expect to find many different study designs exploring the topic of interest, and our research question will be kept broad to capture the range of research on video-based interviews. The methodology for conducting scoping reviews that has been outlined and elaborated on previously will be used [12, 13]. The goals of this scoping review will be to examine the extent, range, and nature of research activity; to summarize and disseminate research findings; and to identify research gaps in the current literature [12].

#### Stage 1: identifying the research question

The purpose of this study will be to review the literature on video-based interviews across the healthcare field to understand the benefits and limitations of video-based interviews, and the barriers associated with transitioning from face-to-face interviews. We will include the perspectives of applicants and interviewees where reported. Outcomes of interest will include environmental cost, financial cost, and the opportunity cost of interviewing. It has been recommended that a broad research question linked to a clearly articulated scope of inquiry be used [13]. Thus, our research question, linked to the aforementioned purpose, target population, and outcomes of interest, is as follows:

*What are the benefits and limitations of video-based interviews for both applicants and programs across healthcare fields, and what are the perceived barriers associated with transitioning away from face-to-face interviews?*

#### Stage 2: identifying relevant studies

A search strategy has been developed by the authors in consultation with an academic health sciences librarian. A combination of Medical Subject Headings (MeSH) and free text was used. The search strategy was developed in light of our research question to keep the search broad, yet focused on the purpose of our scoping review. The full search strategy can be found in Additional File 1. This will be used as is, or with slight modifications, with the following databases: MEDLINE, Embase, PsycInfo, and Cochrane Central.

The results of our search strategy will be checked with relevant references to ensure that we have captured all relevant studies. The grey literature will be searched using Google and OpenGrey to identify relevant conference abstracts or presentations. Authors of any potentially relevant studies will be contacted should more information be required. Citation searches will be performed to ensure that all relevant studies are captured. The reference list of all studies selected for inclusion in this scoping review will also be reviewed.

#### Stage 3: study selection

Inclusion and exclusion criteria have been developed to select the relevant studies from our search, based on recommendations that strict inclusion criteria be utilized during the study selection phase [12]. Our inclusion criteria will be reviewed following the screening process to ensure that selected studies are focused on our research question. The inclusion criteria, listed below, outline the type of study design, participant population, type of intervention, and outcomes of interest.

##### Inclusion criteria

The following are the inclusion criteria:

- Involve applicants interviewing via video-based and/or face-to-face interviews
- Involve applicants applying to medical school, residency, fellowship programs, dentistry, pharmacy, nursing, or other healthcare-related fields
- Any study design that involves the implementation of video-based interviews
- Any method of data analysis, including quantitative and qualitative studies
- Assess any outcome of interest including financial costs, environmental impact, or time invested

##### Exclusion criteria

The following are the exclusion criteria:

- Editorials or expert opinions that do not describe a particular video-based interview that was implemented
- Studies that are not published in English or French

Our search strategy was run on March 31, 2020, after which it has been agreed that we would not include any more studies in the analysis. Mendeley and Microsoft Excel will be used for the study selection process (Mendeley Desktop, version 1.19.4; Microsoft Excel, version 16.35). Two reviewers will apply inclusion and exclusion criteria to all the citations based on the title and abstract. Reviewers will meet at the beginning, midpoint, and final stages of the title and abstract review process to discuss any challenges or uncertainties related to the study selection and to go back and refine the search strategy if needed. Any discrepancies among the two reviewers following title/abstract review will be discussed with a third independent reviewer. Cohen’s kappa statistic will be determined following the independent title and abstract review to ensure inter-rater reliability before proceeding. Cohen’s kappa statistic of *K* > 0.8 (strong level of agreement) will be considered adequate to proceed [14].

Next, the two reviewers will independently review the full text of the articles that met the inclusion criteria based on their title/abstract. The full text will also be reviewed if the relevance of a study is unclear from merely the title/abstract. Reasons for excluding studies after the full-text review will be documented. Where disagreements occur, the third reviewer will be consulted to make the final decision regarding inclusion. The study selection process will be summarized using a PRISMA flow diagram [15].

#### Stage 4: charting the data

DistillerSR (Evidence Partners, Ottawa, Canada) will be used for the data extraction process. A standardized form will be used for data extraction. The form will first be tested by both reviewers on ten of the included studies to determine whether the data extraction approach is consistent with the research question and purpose. The data extraction form will be modified as needed, as we expect to find studies of differing methodologies. Data extraction will then be performed by one reviewer and verified by the second reviewer. Data that will be charted will include the authors, year of publication, study location, study population, sample size, intervention type, comparator (if any), study aims, methodology, outcome measures, and important results.

#### Stage 5: collating, summarizing, and reporting results

Given that we expect varying methodologies and study populations across the different studies that will be included in this scoping review, the analysis of the results will depend on the type of data that is gathered. We suspect that the data analysis will include both a quantitative component to summarize the frequency/demographics of studies and study populations and a qualitative component to outline common themes regarding perceived benefits and limitations of video-based interviews. As we are performing a scoping review expecting heterogeneity in the available literature, a quality assessment of the included studies will not be pursued. While this limits the interpretations that we will be able to draw from our results, this is in line with the purpose of our review which is to examine the extent, range, and nature of the current literature of this topic to guide further research [13].

#### Stage 6: consultation

A consultation stage will be included to share our preliminary findings with stakeholders, validate our results, and inform future research [12, 13]. Specifically, the preliminary findings from stage 5 will be discussed with the stakeholders (i.e., surgeons involved in the interview process) to gain additional sources of information and apply their perspectives to our results. This stage will also serve as an initial knowledge transfer mechanism.

## Discussion

Within the framework proposed by Arksey and O’Malley, the goals of this scoping review are to examine the extent, range, and nature or research activity; to summarize and disseminate research findings; and to identify research gaps in the current literature [12]. The purpose of this scoping review is to explore the benefits and limitations of video-based interviews for both applicants and programs in healthcare fields and identify the perceived barriers associated with transitioning away from face-to-face interviews.

While previous literature has explored the role of video-based interviews among applicants to medical school and residency training programs, it has been met with concerns over the lack of rapport and the inability of applicants to truly represent themselves [7–11]. Given the costly nature of face-to-face interviews from an environmental, economic, and opportunistic point of view, it is prudent that the perceived barriers are better understood in order to facilitate practical changes that allow for an effective transition to video-based interviews. As such, this scoping review will allow for a better understanding of the extent of the literature on video-based fellowship interviews, directions for future research, and practical considerations for training programs looking to transition away from costly face-to-face interviews.

Limitations of this scoping review include the heterogeneity of research methodology that we expect to find in the included studies which will impact our choice of data analysis. In addition, due to the nature of a scoping review, an assessment of the quality of the included studies will not be performed.

### Ethics and dissemination

No intervention or patient recruitment will be required for this study, and as such, research ethics board approval is not required. Following study completion, we plan to disseminate our results via publication in a peer-reviewed journal and/or conference presentation.

## Supplementary information

**Additional file 1.** Search Strategy. Full search strategy developed by the authors in conjunction with an academic health sciences librarian that will be used as is, or with database-specific modifications, to conduct our search.

**Additional file 2.** PRISMA-P 2015 Checklist. Completed PRISMA-P checklist as it applies to this scoping review protocol.

## Data Availability

To ensure transparency and reproducibility, all data generated or analyzed during this study will be included in the published scoping review article and/or its supplementary information files. This will include the search strategy, list of included/excluded studies, reasons for study exclusion, and extracted data used in the analysis.
